# Supplementary material for: Structure-based membrane dome mechanism for Piezo mechanosensitivity
Source: eLife. 2017 Dec 12;6:e33660. doi: 10.7554/eLife.33660 (PMC5788504; doi:10.7554/eLife.33660)
Supplement: Reporting standard 1 [file elife-33660-fig2.pdf]

Full wwPDB/EMDataBank EM Map/Model Validation Report (i)

Nov 3, 2017 – 10:35 AM EDT

PDB ID	:	6B3R
EMDB ID:	:	EMD-7042
Title	:	Structure of the mechanosensitive channel Piezo1
Deposited on	:	2017-09-22
Resolution	:	3.80 Å(reported)
Based on PDB ID	:	4RAX

This is a Full wwPDB/EMDataBank EM Map/Model Validation Report.

This report is produced by the wwPDB biocuration pipeline after annotation of the structure.

We welcome your comments at *validation@mail.wwpdb.org*
A user guide is available at
http://wwpdb.org/validation/2016/EMValidationReportHelp
with specific help available everywhere you see the (i) symbol.

MolProbity	:	4.02b-467
Percentile statistics	:	20161228.v01 (using entries in the PDB archive December 28th 2016)
Ideal geometry (proteins)	:	Engh & Huber (2001)
Ideal geometry (DNA, RNA)	:	Parkinson et. al. (1996)
Validation Pipeline (wwPDB-VP)	:	rb-20030345

CONFIDENTIAL VALIDATION REPORT

1 Overall quality at a glance (i)

The following experimental techniques were used to determine the structure:
ELECTRON MICROSCOPY

The reported resolution of this entry is 3.80 Å.

Percentile scores (ranging between 0-100) for global validation metrics of the entry are shown in the following graphic. The table shows the number of entries on which the scores are based.

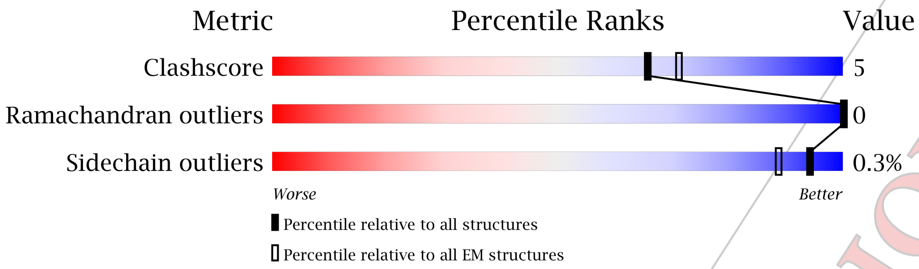

Metric	Whole archive (#Entries)	EM structures (#Entries)
Clashscore	125131	1336
Ramachandran outliers	121729	1120
Sidechain outliers	121581	1026

The table below summarises the geometric issues observed across the polymeric chains. The red, orange, yellow and green segments on the bar indicate the fraction of residues that contain outliers for >=3, 2, 1 and 0 types of geometric quality criteria. A grey segment represents the fraction of residues that are not modelled. The numeric value for each fraction is indicated below the corresponding segment, with a dot representing fractions <=5%

Mol	Chain	Length	Quality of chain
1	A	2547	51% 8% 41%
1	C	2547	51% 7% 41%
1	E	2547	51% 8% 41%
2	B	16	100%
2	D	16	100%
2	F	16	100%

CONFIDENTIAL VALIDATION REPORT

2 Entry composition (i)

There are 2 unique types of molecules in this entry. The entry contains 35718 atoms, of which 0 are hydrogens and 0 are deuteriums.

In the tables below, the AltConf column contains the number of residues with at least one atom in alternate conformation and the Trace column contains the number of residues modelled with at most 2 atoms.

- Molecule 1 is a protein called Piezo-type mechanosensitive ion channel component 1.

Mol	Chain	Residues	Atoms					AltConf	Trace
1	A	1502	Total 11826	C 7739	N 2004	O 2013	S 70	0	0
1	C	1502	Total 11826	C 7739	N 2004	O 2013	S 70	0	0
1	E	1502	Total 11826	C 7739	N 2004	O 2013	S 70	0	0

- Molecule 2 is a protein called Piezo-type mechanosensitive ion channel component 1, unknown fragment.

Mol	Chain	Residues	Atoms				AltConf	Trace
2	B	16	Total 80	C 48	N 16	O 16	0	0
2	D	16	Total 80	C 48	N 16	O 16	0	0
2	F	16	Total 80	C 48	N 16	O 16	0	0

CONFIDENTIAL VALIDATION REPORT

3 Residue-property plots (i)

These plots are drawn for all protein, RNA and DNA chains in the entry. The first graphic for a chain summarises the proportions of the various outlier classes displayed in the second graphic. The second graphic shows the sequence view annotated by issues in geometry. Residues are color-coded according to the number of geometric quality criteria for which they contain at least one outlier: green = 0, yellow = 1, orange = 2 and red = 3 or more. Stretches of 2 or more consecutive residues without any outlier are shown as a green connector. Residues present in the sample, but not in the model, are shown in grey.

- Molecule 1: Piezo-type mechanosensitive ion channel component 1

Chain A:

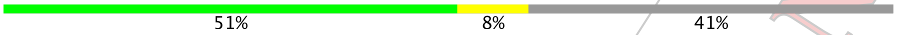

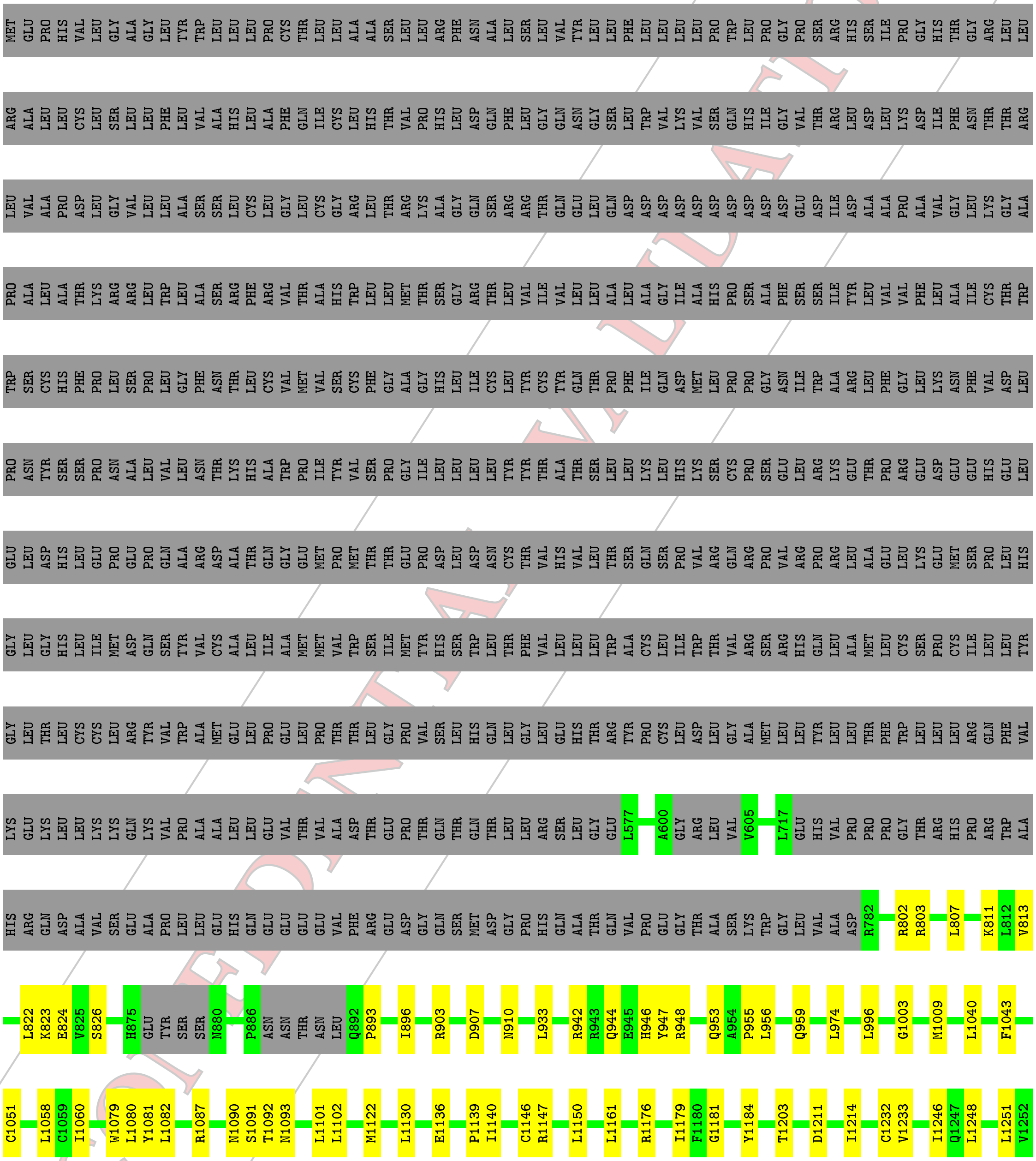

CONFIDENTIAL VALIDATION REPORT

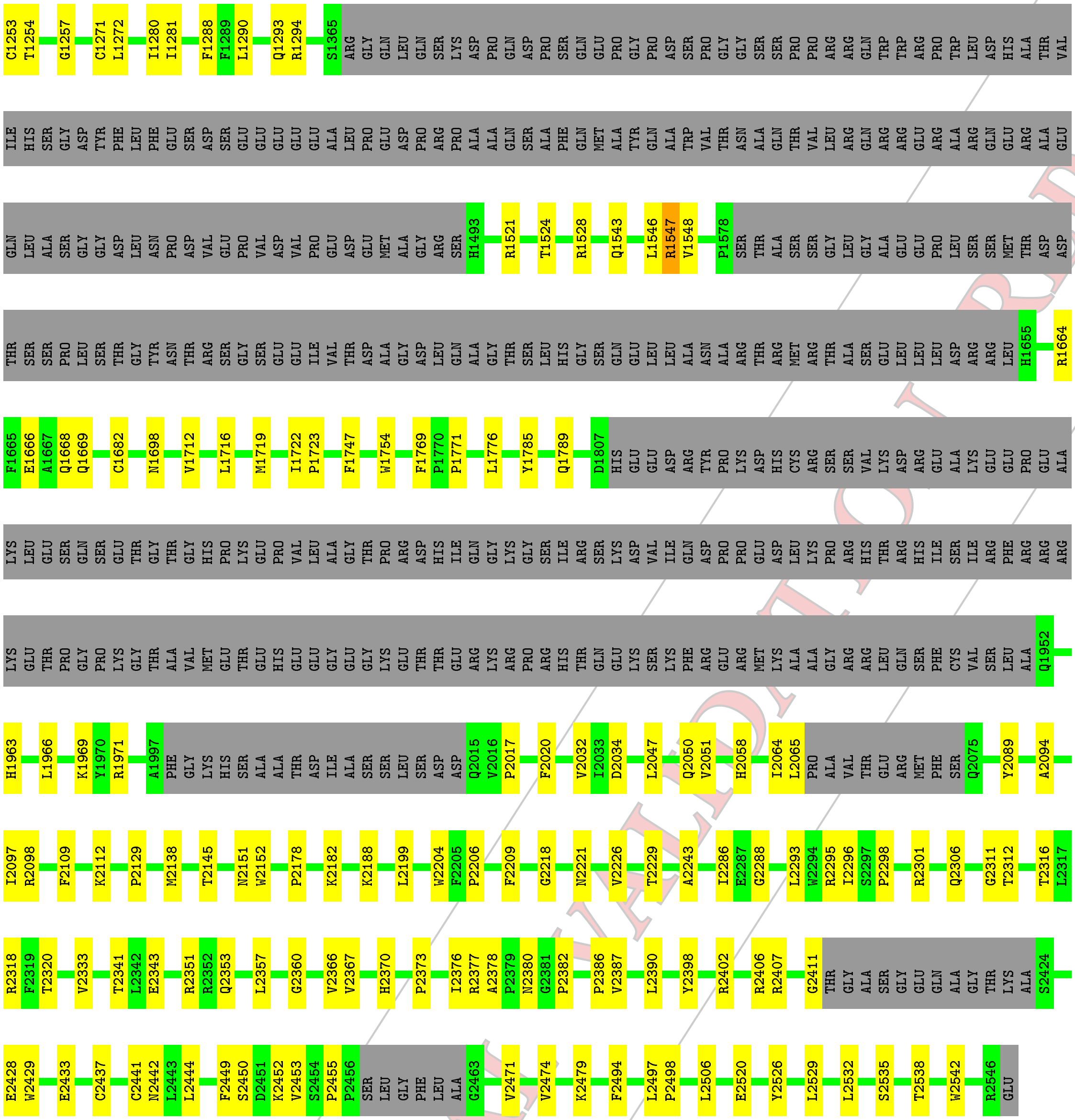

• Molecule 1: Piezo-type mechanosensitive ion channel component 1

Chain C:

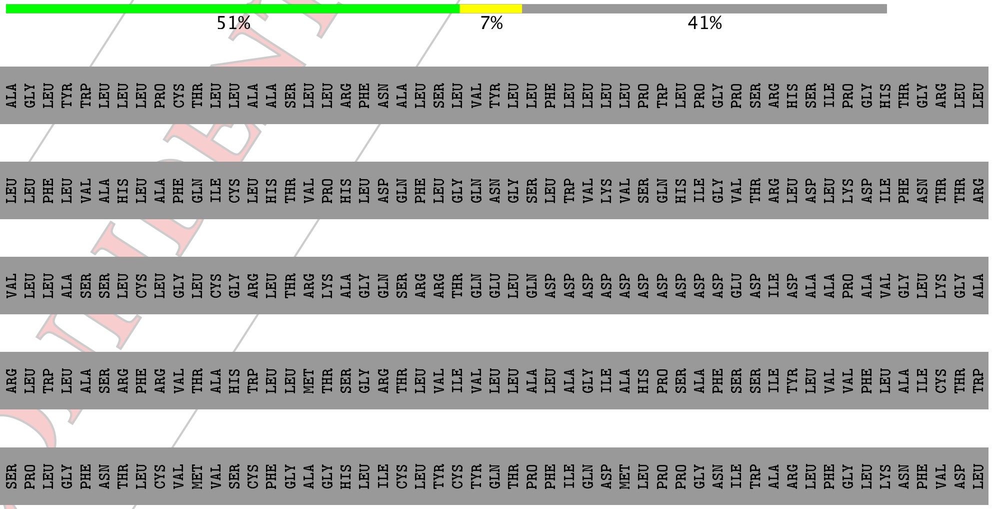

CONFIDENTIAL VALIDATION REPORT

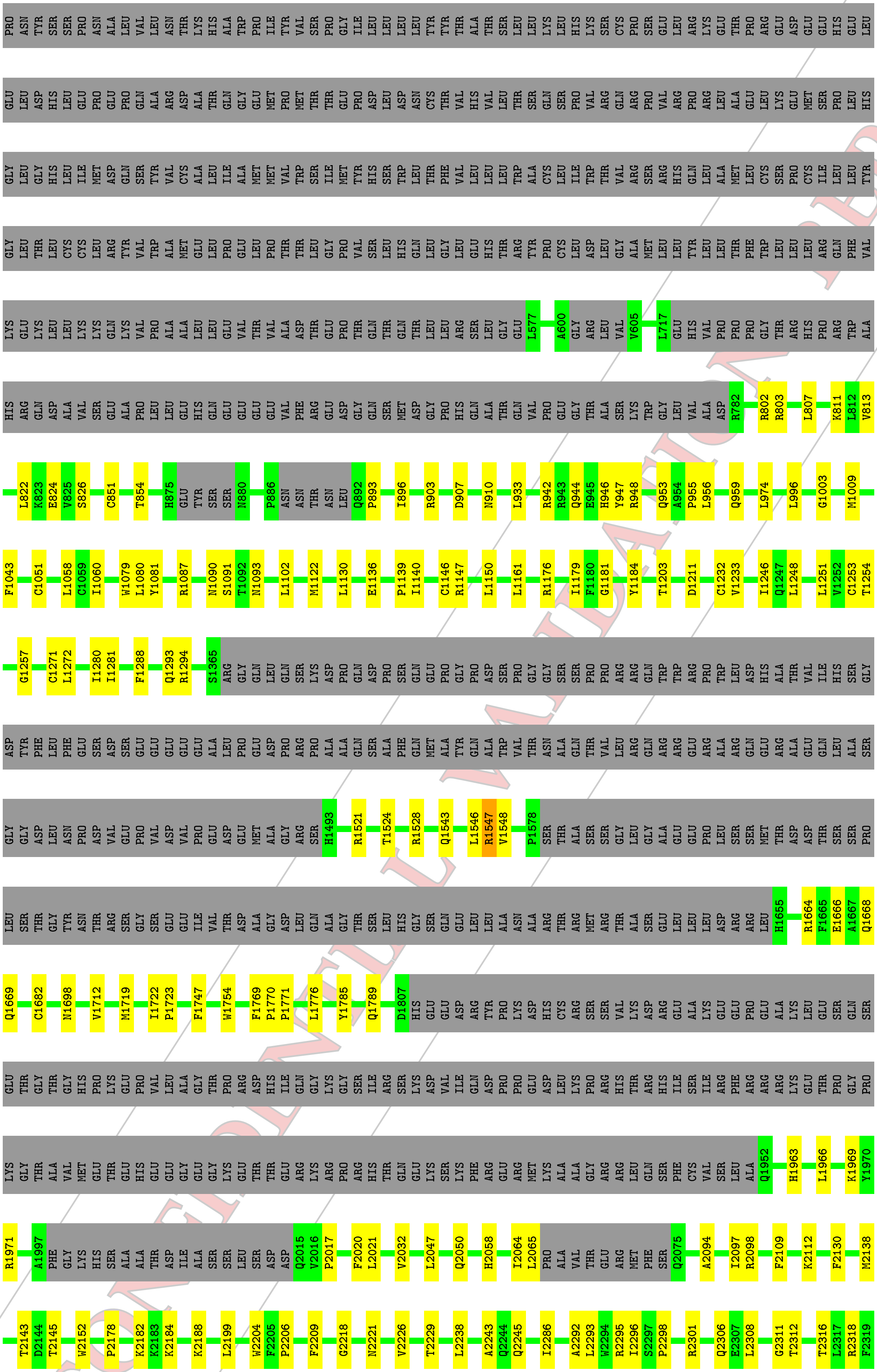

CONFIDENTIAL VALIDATION REPORT

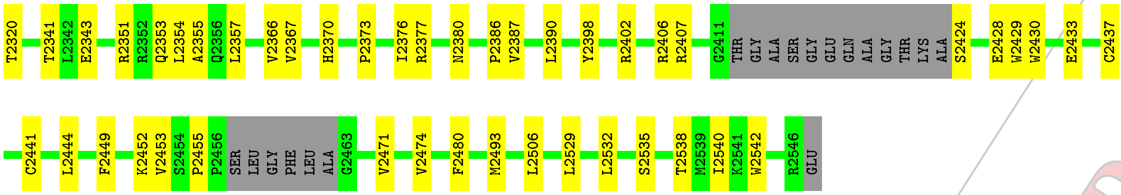

• Molecule 1: Piezo-type mechanosensitive ion channel component 1

Chain E:

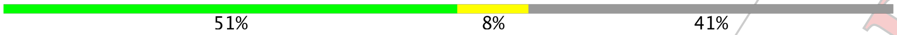

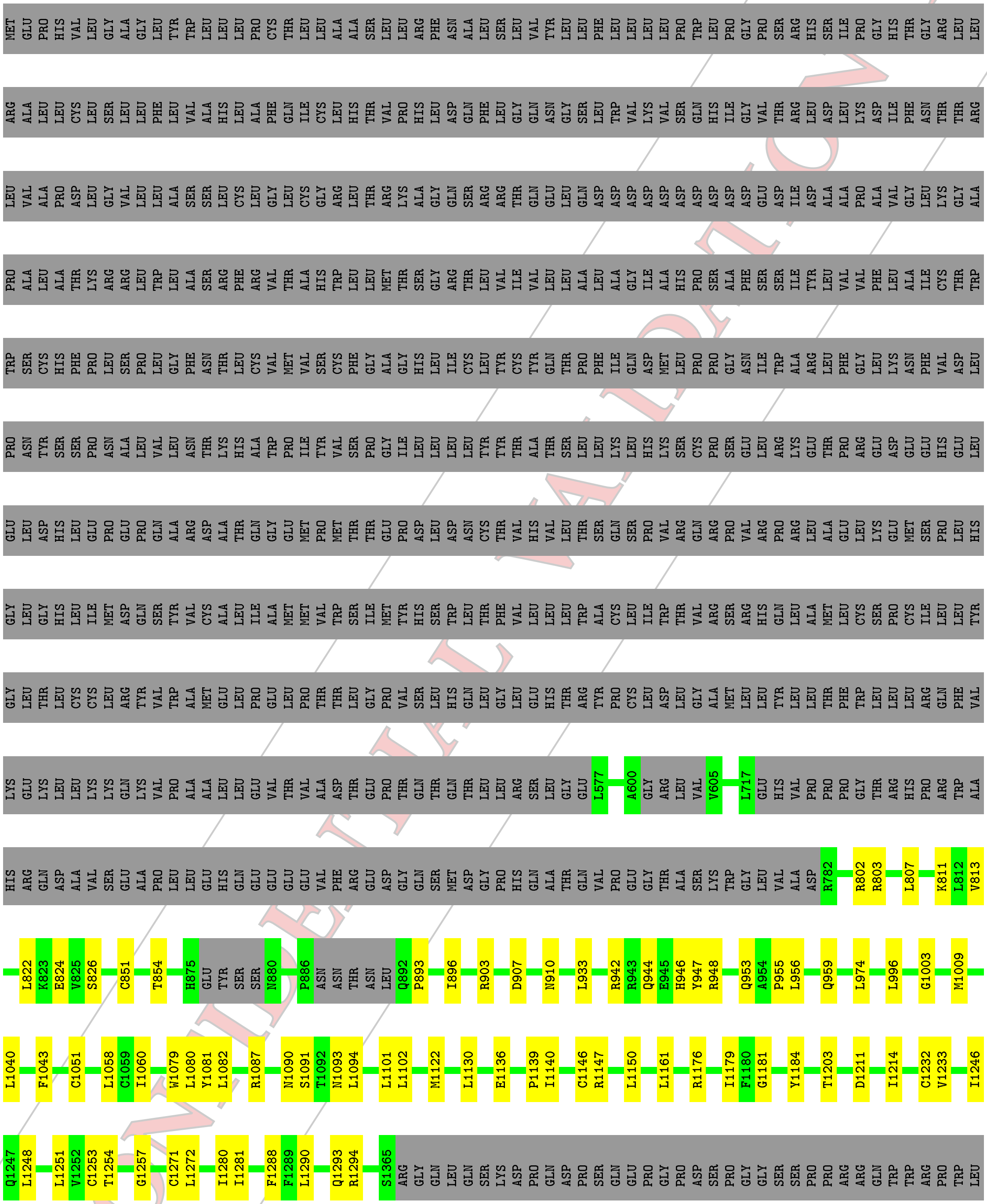

CONFIDENTIAL VALIDATION REPORT

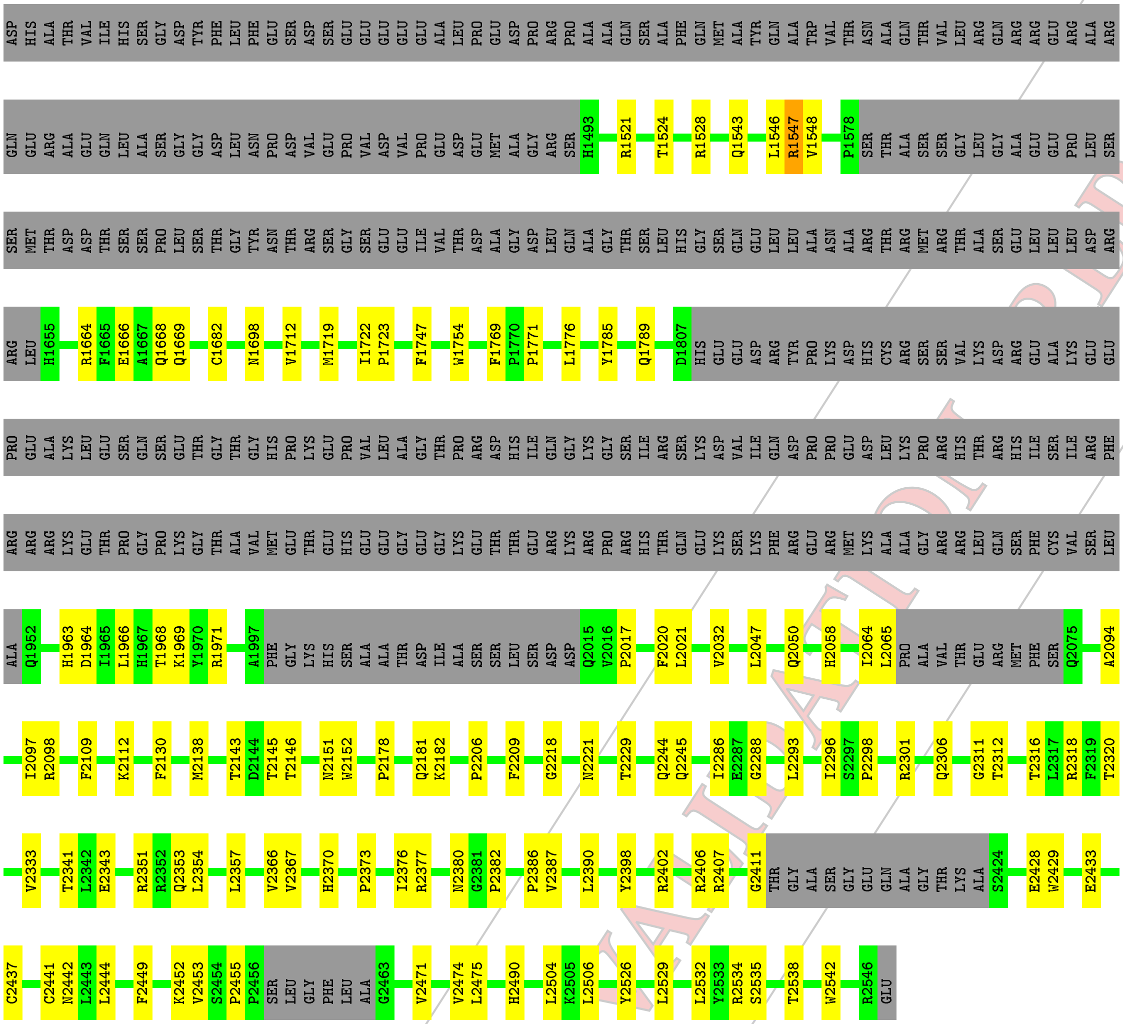

• Molecule 2: Piezo-type mechanosensitive ion channel component 1, unknown fragment

Chain B:

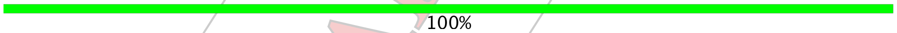

There are no outlier residues recorded for this chain.

• Molecule 2: Piezo-type mechanosensitive ion channel component 1, unknown fragment

Chain D:

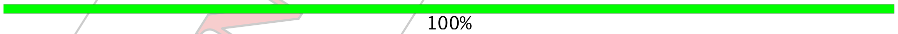

There are no outlier residues recorded for this chain.

• Molecule 2: Piezo-type mechanosensitive ion channel component 1, unknown fragment

Chain F:

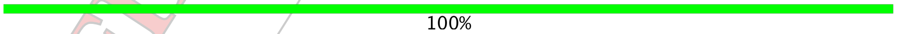

There are no outlier residues recorded for this chain.

CONFIDENTIAL VALIDATION REPORT

4 Experimental information (i)

Property	Value	Source
Reconstruction method	SINGLE PARTICLE	Depositor
Imposed symmetry	POINT, C1	Depositor
Number of particles used	277548	Depositor
Resolution determination method	FSC 0.143 CUT-OFF	Depositor
CTF correction method	PHASE FLIPPING AND AMPLITUDE CORRECTION	Depositor
Microscope	FEI TITAN KRIOS	Depositor
Voltage (kV)	300	Depositor
Electron dose $(e^-/\text{Å}^2)$	47	Depositor
Minimum defocus (nm)	Not provided	Depositor
Maximum defocus (nm)	Not provided	Depositor
Magnification	Not provided	Depositor
Image detector	GATAN K2 SUMMIT (4k x 4k)	Depositor

CONFIDENTIAL VALIDATION REPORT

5 Model quality (i)

5.1 Standard geometry (i)

The Z score for a bond length (or angle) is the number of standard deviations the observed value is removed from the expected value. A bond length (or angle) with $|Z| > 5$ is considered an outlier worth inspection. RMSZ is the root-mean-square of all Z scores of the bond lengths (or angles).

Mol	Chain	Bond lengths		Bond angles	
		RMSZ	#|Z| >2	RMSZ	#|Z| >2
1	A	0.35	0/12117	0.59	0/16473
1	C	0.35	0/12117	0.59	0/16473
1	E	0.34	0/12117	0.59	0/16473
All	All	0.35	0/36351	0.59	0/49419

There are no bond length outliers.

There are no bond angle outliers.

There are no chirality outliers.

There are no planarity outliers.

5.2 Too-close contacts (i)

In the following table, the Non-H and H(model) columns list the number of non-hydrogen atoms and hydrogen atoms in the chain respectively. The H(added) column lists the number of hydrogen atoms added and optimized by MolProbity. The Clashes column lists the number of clashes within the asymmetric unit, whereas Symm-Clashes lists symmetry related clashes.

Mol	Chain	Non-H	H(model)	H(added)	Clashes	Symm-Clashes
1	A	11826	0	11563	118	0
1	C	11826	0	11563	119	0
1	E	11826	0	11563	122	0
2	B	80	0	19	0	0
2	D	80	0	19	0	0
2	F	80	0	19	0	0
All	All	35718	0	34746	333	0

The all-atom clashscore is defined as the number of clashes found per 1000 atoms (including hydrogen atoms). The all-atom clashscore for this structure is 5.

All (333) close contacts within the same asymmetric unit are listed below, sorted by their clash magnitude.

CONFIDENTIAL VALIDATION REPORT

Atom-1	Atom-2	Interatomic distance (Å)	Clash overlap (Å)
1:C:2050:GLN:HE22	1:C:2094:ALA:HB2	1.57	0.69
1:E:2050:GLN:HE22	1:E:2094:ALA:HB2	1.57	0.69
1:A:2050:GLN:HE22	1:A:2094:ALA:HB2	1.57	0.69
1:A:1161:LEU:O	1:A:1293:GLN:NE2	2.29	0.66
1:E:1161:LEU:O	1:E:1293:GLN:NE2	2.29	0.65
1:C:2406:ARG:HB3	1:C:2429:TRP:HB2	1.78	0.65
1:C:1161:LEU:O	1:C:1293:GLN:NE2	2.29	0.65
1:E:2406:ARG:HB3	1:E:2429:TRP:HB2	1.78	0.65
1:A:2367:VAL:HG22	1:A:2402:ARG:HG2	1.80	0.64
1:A:2406:ARG:HB3	1:A:2429:TRP:HB2	1.78	0.64
1:E:2229:THR:HG1	1:E:2320:THR:HG1	1.44	0.63
1:E:2367:VAL:HG22	1:E:2402:ARG:HG2	1.80	0.63
1:A:1698:ASN:ND2	1:A:1789:GLN:OE1	2.32	0.63
1:C:2367:VAL:HG22	1:C:2402:ARG:HG2	1.80	0.63
1:A:2286:ILE:HB	1:A:2444:LEU:HB2	1.81	0.62
1:A:2229:THR:HG1	1:A:2320:THR:HG1	1.47	0.62
1:C:1698:ASN:ND2	1:C:1789:GLN:OE1	2.32	0.62
1:C:2188:LYS:NZ	1:E:2143:THR:O	2.30	0.62
1:C:2296:ILE:O	1:E:2429:TRP:NE1	2.33	0.62
1:A:2296:ILE:HD12	1:A:2301:ARG:HE	1.65	0.62
1:C:2286:ILE:HB	1:C:2444:LEU:HB2	1.81	0.61
1:E:2047:LEU:HD12	1:E:2097:ILE:HD12	1.82	0.61
1:E:1698:ASN:ND2	1:E:1789:GLN:OE1	2.32	0.61
1:E:2286:ILE:HB	1:E:2444:LEU:HB2	1.81	0.61
1:C:1698:ASN:HB3	1:C:1785:TYR:HB2	1.82	0.61
1:A:2178:PRO:HD2	1:A:2182:LYS:HD2	1.83	0.61
1:C:2295:ARG:NH1	1:E:2293:LEU:O	2.33	0.61
1:C:2296:ILE:HD12	1:C:2301:ARG:HE	1.65	0.61
1:A:1698:ASN:HB3	1:A:1785:TYR:HB2	1.82	0.60
1:E:2296:ILE:HD12	1:E:2301:ARG:HE	1.65	0.60
1:C:2178:PRO:HD2	1:C:2182:LYS:HD2	1.83	0.60
1:C:1543:GLN:O	1:C:1547:ARG:NH1	2.35	0.60
1:A:2295:ARG:NH1	1:C:2293:LEU:O	2.35	0.60
1:C:1666:GLU:HA	1:C:1669:GLN:HE21	1.66	0.60
1:E:1698:ASN:HB3	1:E:1785:TYR:HB2	1.82	0.60
1:E:1543:GLN:O	1:E:1547:ARG:NH1	2.35	0.59
1:E:1666:GLU:HA	1:E:1669:GLN:HE21	1.66	0.59
1:C:2047:LEU:HD12	1:C:2097:ILE:HD12	1.82	0.59
1:A:1543:GLN:O	1:A:1547:ARG:NH1	2.35	0.59
1:E:2178:PRO:HD2	1:E:2182:LYS:HD2	1.83	0.59
1:A:2047:LEU:HD12	1:A:2097:ILE:HD12	1.83	0.59
1:E:2377:ARG:HA	1:E:2449:PHE:HB2	1.85	0.59

Continued on next page...

CONFIDENTIAL VALIDATION REPORT

Continued from previous page...

Atom-1	Atom-2	Interatomic distance (Å)	Clash overlap (Å)
1:E:2206:PRO:HA	1:E:2209:PHE:HB3	1.84	0.59
1:A:2206:PRO:HA	1:A:2209:PHE:HB3	1.84	0.59
1:A:2377:ARG:HA	1:A:2449:PHE:HB2	1.85	0.59
1:E:2370:HIS:ND1	1:E:2398:TYR:O	2.35	0.59
1:C:1146:CYS:SG	1:C:1147:ARG:N	2.76	0.59
1:C:2229:THR:HG1	1:C:2320:THR:HG1	1.49	0.59
1:C:1051:CYS:SG	1:C:1081:TYR:OH	2.60	0.58
1:A:1146:CYS:SG	1:A:1147:ARG:N	2.76	0.58
1:C:2370:HIS:ND1	1:C:2398:TYR:O	2.35	0.58
1:E:2377:ARG:HG3	1:E:2387:VAL:HG22	1.85	0.58
1:C:2206:PRO:HA	1:C:2209:PHE:HB3	1.84	0.58
1:C:1547:ARG:HG2	1:C:1548:VAL:HG13	1.86	0.58
1:E:1524:THR:HG21	1:E:1682:CYS:HA	1.86	0.58
1:A:1666:GLU:HA	1:A:1669:GLN:HE21	1.66	0.58
1:E:1146:CYS:SG	1:E:1147:ARG:N	2.76	0.58
1:A:1722:ILE:HD11	1:A:1966:LEU:HD23	1.86	0.58
1:C:2377:ARG:HG3	1:C:2387:VAL:HG22	1.85	0.58
1:E:1722:ILE:HD11	1:E:1966:LEU:HD23	1.86	0.57
1:A:2377:ARG:HG3	1:A:2387:VAL:HG22	1.85	0.57
1:C:2377:ARG:HA	1:C:2449:PHE:HB2	1.84	0.57
1:A:1524:THR:HG21	1:A:1682:CYS:HA	1.86	0.57
1:E:907:ASP:HB3	1:E:910:ASN:HB2	1.86	0.57
1:E:948:ARG:HE	1:E:953:GLN:HB2	1.70	0.57
1:A:2370:HIS:ND1	1:A:2398:TYR:O	2.35	0.57
1:C:948:ARG:HE	1:C:953:GLN:HB2	1.70	0.57
1:C:1722:ILE:HD11	1:C:1966:LEU:HD23	1.86	0.57
1:E:2316:THR:HG22	1:E:2343:GLU:HG2	1.87	0.57
1:A:1719:MET:O	1:A:2098:ARG:NH1	2.38	0.57
1:A:1547:ARG:HG2	1:A:1548:VAL:HG13	1.86	0.56
1:A:948:ARG:HE	1:A:953:GLN:HB2	1.70	0.56
1:C:1524:THR:HG21	1:C:1682:CYS:HA	1.86	0.56
1:A:2032:VAL:HA	1:A:2138:MET:HE1	1.87	0.56
1:A:1664:ARG:O	1:A:1668:GLN:NE2	2.39	0.56
1:A:1966:LEU:HD13	1:A:2047:LEU:HD21	1.87	0.56
1:A:907:ASP:HB3	1:A:910:ASN:HB2	1.86	0.56
1:C:1719:MET:O	1:C:2098:ARG:NH1	2.38	0.56
1:E:2032:VAL:HA	1:E:2138:MET:HE1	1.88	0.56
1:E:1719:MET:O	1:E:2098:ARG:NH1	2.38	0.56
1:E:1547:ARG:HG2	1:E:1548:VAL:HG13	1.86	0.56
1:E:1664:ARG:O	1:E:1668:GLN:NE2	2.39	0.56
1:A:2437:CYS:HB3	1:A:2441:CYS:HA	1.88	0.55

Continued on next page...

CONFIDENTIAL VALIDATION REPORT

Continued from previous page...

Atom-1	Atom-2	Interatomic distance (Å)	Clash overlap (Å)
1:C:1664:ARG:O	1:C:1668:GLN:NE2	2.39	0.55
1:C:2316:THR:HG22	1:C:2343:GLU:HG2	1.87	0.55
1:A:2316:THR:HG22	1:A:2343:GLU:HG2	1.87	0.55
1:C:907:ASP:HB3	1:C:910:ASN:HB2	1.86	0.55
1:E:2437:CYS:HB3	1:E:2441:CYS:HA	1.87	0.55
1:C:2424:SER:HB2	1:E:2411:GLY:HA3	1.88	0.55
1:E:1966:LEU:HD13	1:E:2047:LEU:HD21	1.87	0.55
1:C:1966:LEU:HD13	1:C:2047:LEU:HD21	1.87	0.55
1:C:2032:VAL:HA	1:C:2138:MET:HE1	1.86	0.55
1:C:2437:CYS:HB3	1:C:2441:CYS:HA	1.88	0.55
1:C:803:ARG:HE	1:C:955:PRO:HG2	1.72	0.55
1:E:1051:CYS:SG	1:E:1081:TYR:OH	2.60	0.55
1:A:956:LEU:HD22	1:A:1122:MET:HG3	1.89	0.54
1:E:1051:CYS:HG	1:E:1081:TYR:HH	1.52	0.54
1:E:956:LEU:HD22	1:E:1122:MET:HG3	1.89	0.54
1:A:2357:LEU:O	1:A:2407:ARG:NH2	2.41	0.54
1:A:803:ARG:HE	1:A:955:PRO:HG2	1.72	0.54
1:E:2357:LEU:O	1:E:2407:ARG:NH2	2.41	0.54
1:A:813:VAL:HG22	1:A:933:LEU:HB3	1.90	0.53
1:C:2199:LEU:HD21	1:E:2130:PHE:HB3	1.90	0.53
1:C:813:VAL:HG22	1:C:933:LEU:HB3	1.90	0.53
1:E:803:ARG:HE	1:E:955:PRO:HG2	1.72	0.53
1:C:1090:ASN:ND2	1:C:1093:ASN:OD1	2.42	0.53
1:C:956:LEU:HD22	1:C:1122:MET:HG3	1.89	0.53
1:A:2221:ASN:ND2	1:A:2380:ASN:OD1	2.42	0.53
1:C:2221:ASN:ND2	1:C:2380:ASN:OD1	2.42	0.53
1:C:2306:GLN:O	1:C:2311:GLY:N	2.39	0.53
1:C:2301:ARG:NH2	1:E:2411:GLY:O	2.42	0.53
1:C:2298:PRO:HG2	1:E:2406:ARG:HD3	1.89	0.53
1:E:813:VAL:HG22	1:E:933:LEU:HB3	1.90	0.53
1:C:2357:LEU:O	1:C:2407:ARG:NH2	2.41	0.52
1:E:1090:ASN:ND2	1:E:1093:ASN:OD1	2.42	0.52
1:A:1090:ASN:ND2	1:A:1093:ASN:OD1	2.42	0.52
1:A:1003:GLY:HA2	1:A:1280:ILE:HD13	1.92	0.52
1:C:2402:ARG:N	1:C:2433:GLU:O	2.42	0.52
1:E:2306:GLN:O	1:E:2311:GLY:N	2.39	0.52
1:E:2221:ASN:ND2	1:E:2380:ASN:OD1	2.42	0.52
1:E:1003:GLY:HA2	1:E:1280:ILE:HD13	1.92	0.52
1:C:1003:GLY:HA2	1:C:1280:ILE:HD13	1.92	0.51
1:A:1723:PRO:HD3	1:A:1969:LYS:HE3	1.93	0.51
1:A:2306:GLN:O	1:A:2311:GLY:N	2.39	0.51

Continued on next page...

CONFIDENTIAL VALIDATION REPORT

Continued from previous page...

Atom-1	Atom-2	Interatomic distance (Å)	Clash overlap (Å)
1:C:1723:PRO:HD3	1:C:1969:LYS:HE3	1.93	0.51
1:A:1058:LEU:HB3	1:A:1060:ILE:HG22	1.93	0.51
1:E:1723:PRO:HD3	1:E:1969:LYS:HE3	1.93	0.51
1:C:1058:LEU:HB3	1:C:1060:ILE:HG22	1.93	0.50
1:E:1058:LEU:HB3	1:E:1060:ILE:HG22	1.93	0.50
1:E:1136:GLU:HB3	1:E:1546:LEU:HD21	1.94	0.50
1:A:824:GLU:HG3	1:A:826:SER:HB3	1.93	0.50
1:A:2402:ARG:N	1:A:2433:GLU:O	2.42	0.50
1:C:824:GLU:HG3	1:C:826:SER:HB3	1.93	0.50
1:E:824:GLU:HG3	1:E:826:SER:HB3	1.93	0.50
1:A:1130:LEU:HD12	1:A:1203:THR:HG21	1.93	0.50
1:C:1136:GLU:HB3	1:C:1546:LEU:HD21	1.94	0.50
1:C:1130:LEU:HD12	1:C:1203:THR:HG21	1.93	0.49
1:A:2411:GLY:O	1:E:2301:ARG:NH2	2.44	0.49
1:E:2373:PRO:HG2	1:E:2376:ILE:HD11	1.94	0.49
1:A:2373:PRO:HG2	1:A:2376:ILE:HD11	1.94	0.49
1:E:1754:TRP:HB2	1:E:1769:PHE:HZ	1.77	0.49
1:A:1136:GLU:HB3	1:A:1546:LEU:HD21	1.94	0.49
1:E:2017:PRO:HA	1:E:2020:PHE:HD2	1.78	0.49
1:A:2376:ILE:HA	1:A:2386:PRO:HA	1.95	0.49
1:E:1009:MET:HB3	1:E:1043:PHE:HZ	1.78	0.49
1:E:942:ARG:O	1:E:946:HIS:ND1	2.45	0.49
1:E:2376:ILE:HA	1:E:2386:PRO:HA	1.95	0.49
1:E:1130:LEU:HD12	1:E:1203:THR:HG21	1.93	0.48
1:C:2376:ILE:HA	1:C:2386:PRO:HA	1.95	0.48
1:E:893:PRO:HD2	1:E:896:ILE:HD13	1.95	0.48
1:A:1754:TRP:HB2	1:A:1769:PHE:HZ	1.77	0.48
1:C:2373:PRO:HG2	1:C:2376:ILE:HD11	1.94	0.48
1:C:2387:VAL:HG12	1:C:2390:LEU:H	1.78	0.48
1:C:893:PRO:HD2	1:C:896:ILE:HD13	1.95	0.48
1:A:2506:LEU:HD22	1:A:2529:LEU:HG	1.96	0.48
1:C:2218:GLY:HA2	1:C:2452:LYS:HD3	1.96	0.48
1:E:2402:ARG:N	1:E:2433:GLU:O	2.42	0.48
1:A:2017:PRO:HA	1:A:2020:PHE:HD2	1.78	0.48
1:A:2199:LEU:HD21	1:C:2130:PHE:HB3	1.96	0.48
1:A:2218:GLY:HA2	1:A:2452:LYS:HD3	1.96	0.48
1:C:1754:TRP:HB2	1:C:1769:PHE:HZ	1.78	0.48
1:E:1246:ILE:HA	1:E:1251:LEU:HD12	1.96	0.48
1:C:2506:LEU:HD22	1:C:2529:LEU:HG	1.96	0.48
1:A:1009:MET:HB3	1:A:1043:PHE:HZ	1.78	0.47
1:A:1139:PRO:HG2	1:A:1140:ILE:HD12	1.96	0.47

Continued on next page...

CONFIDENTIAL VALIDATION REPORT

Continued from previous page...

Atom-1	Atom-2	Interatomic distance (Å)	Clash overlap (Å)
1:A:893:PRO:HD2	1:A:896:ILE:HD13	1.95	0.47
1:C:1139:PRO:HG2	1:C:1140:ILE:HD12	1.96	0.47
1:C:974:LEU:HD23	1:C:1150:LEU:HD23	1.96	0.47
1:A:974:LEU:HD23	1:A:1150:LEU:HD23	1.96	0.47
1:E:2387:VAL:HG12	1:E:2390:LEU:H	1.78	0.47
1:A:1246:ILE:HA	1:A:1251:LEU:HD12	1.96	0.47
1:A:2387:VAL:HG12	1:A:2390:LEU:H	1.78	0.47
1:A:2295:ARG:HD3	1:C:2292:ALA:HA	1.97	0.47
1:E:2532:LEU:HD11	1:E:2542:TRP:CD1	2.50	0.47
1:A:2532:LEU:HD11	1:A:2542:TRP:CD1	2.50	0.47
1:C:1009:MET:HB3	1:C:1043:PHE:HZ	1.78	0.47
1:C:2017:PRO:HA	1:C:2020:PHE:HD2	1.78	0.47
1:E:1271:CYS:SG	1:E:1272:LEU:N	2.88	0.47
1:A:1051:CYS:SG	1:A:1081:TYR:OH	2.60	0.47
1:E:974:LEU:HD23	1:E:1150:LEU:HD23	1.96	0.47
1:C:1246:ILE:HA	1:C:1251:LEU:HD12	1.96	0.47
1:E:2298:PRO:HA	1:E:2301:ARG:HB2	1.97	0.47
1:E:1139:PRO:HG2	1:E:1140:ILE:HD12	1.96	0.47
1:E:2506:LEU:HD22	1:E:2529:LEU:HG	1.96	0.47
1:C:1271:CYS:SG	1:C:1272:LEU:N	2.88	0.46
1:E:2218:GLY:HA2	1:E:2452:LYS:HD3	1.96	0.46
1:A:1271:CYS:SG	1:A:1272:LEU:N	2.88	0.46
1:A:2298:PRO:HA	1:A:2301:ARG:HB2	1.97	0.46
1:E:1712:VAL:HG22	1:E:2058:HIS:HD1	1.81	0.46
1:A:1712:VAL:HG22	1:A:2058:HIS:HD1	1.81	0.46
1:E:1181:GLY:HA2	1:E:1184:TYR:HD2	1.81	0.46
1:A:1181:GLY:HA2	1:A:1184:TYR:HD2	1.81	0.46
1:C:1181:GLY:HA2	1:C:1184:TYR:HD2	1.81	0.46
1:A:2318:ARG:NH2	1:C:2245:GLN:O	2.49	0.46
1:C:2532:LEU:HD11	1:C:2542:TRP:CD1	2.50	0.46
1:A:2145:THR:HG21	1:A:2152:TRP:HZ3	1.81	0.46
1:C:2453:VAL:HG12	1:C:2455:PRO:HD3	1.98	0.46
1:A:947:TYR:HD2	1:A:953:GLN:HE21	1.64	0.46
1:C:807:LEU:HD13	1:C:959:GLN:HG3	1.98	0.46
1:E:947:TYR:HD2	1:E:953:GLN:HE21	1.64	0.46
1:A:807:LEU:HD13	1:A:959:GLN:HG3	1.98	0.45
1:C:1179:ILE:HD11	1:C:1248:LEU:HD12	1.98	0.45
1:A:942:ARG:O	1:A:946:HIS:ND1	2.45	0.45
1:C:2145:THR:HG21	1:C:2152:TRP:HZ3	1.81	0.45
1:C:2540:ILE:HD11	1:E:2535:SER:HB3	1.99	0.45
1:C:1747:PHE:HB2	1:C:1776:LEU:HD12	1.99	0.45

Continued on next page...

CONFIDENTIAL VALIDATION REPORT

Continued from previous page...

Atom-1	Atom-2	Interatomic distance (Å)	Clash overlap (Å)
1:C:2298:PRO:HA	1:C:2301:ARG:HB2	1.97	0.45
1:A:1747:PHE:HB2	1:A:1776:LEU:HD12	1.99	0.45
1:C:811:LYS:HD2	1:C:1102:LEU:HD21	1.99	0.45
1:E:1747:PHE:HB2	1:E:1776:LEU:HD12	1.99	0.45
1:C:1246:ILE:HG23	1:C:1251:LEU:HB2	1.99	0.45
1:C:947:TYR:HD2	1:C:953:GLN:HE21	1.64	0.45
1:A:1176:ARG:HH22	1:A:1281:ILE:HD12	1.82	0.45
1:C:1232:CYS:SG	1:C:1233:VAL:N	2.90	0.45
1:E:2318:ARG:HG2	1:E:2341:THR:HG22	1.98	0.45
1:A:1246:ILE:HG23	1:A:1251:LEU:HB2	1.99	0.45
1:A:2318:ARG:HG2	1:A:2341:THR:HG22	1.98	0.45
1:C:1712:VAL:HG22	1:C:2058:HIS:HD1	1.81	0.45
1:A:1232:CYS:SG	1:A:1233:VAL:N	2.90	0.45
1:E:1246:ILE:HG23	1:E:1251:LEU:HB2	1.99	0.44
1:E:811:LYS:HD2	1:E:1102:LEU:HD21	1.99	0.44
1:A:1771:PRO:HB3	1:A:1776:LEU:HB2	1.99	0.44
1:E:1179:ILE:HD11	1:E:1248:LEU:HD12	1.98	0.44
1:E:807:LEU:HD13	1:E:959:GLN:HG3	1.98	0.44
1:A:2520:GLU:HG3	1:E:2181:GLN:HG3	1.99	0.44
1:C:2312:THR:HA	1:C:2351:ARG:HH12	1.82	0.44
1:E:2145:THR:HG21	1:E:2152:TRP:HZ3	1.81	0.44
1:A:1079:TRP:HE3	1:A:1080:LEU:HD12	1.83	0.44
1:A:1179:ILE:HD11	1:A:1248:LEU:HD12	1.98	0.44
1:E:2453:VAL:HG12	1:E:2455:PRO:HD3	1.98	0.44
1:A:2109:PHE:HA	1:A:2112:LYS:HE2	2.00	0.44
1:A:811:LYS:HD2	1:A:1102:LEU:HD21	1.99	0.44
1:C:2318:ARG:HG2	1:C:2341:THR:HG22	1.98	0.44
1:E:1232:CYS:SG	1:E:1233:VAL:N	2.90	0.44
1:A:2453:VAL:HG12	1:A:2455:PRO:HD3	1.98	0.44
1:E:1079:TRP:HE3	1:E:1080:LEU:HD12	1.83	0.44
1:A:2494:PHE:HE2	1:E:2490:HIS:HA	1.81	0.44
1:A:822:LEU:HD22	1:A:1091:SER:HB2	2.00	0.44
1:C:1176:ARG:HH22	1:C:1281:ILE:HD12	1.82	0.44
1:C:1771:PRO:HB3	1:C:1776:LEU:HB2	1.99	0.44
1:A:2312:THR:HA	1:A:2351:ARG:HH12	1.82	0.43
1:C:2109:PHE:HA	1:C:2112:LYS:HE2	2.00	0.43
1:E:1771:PRO:HB3	1:E:1776:LEU:HB2	1.99	0.43
1:A:2494:PHE:CE2	1:E:2490:HIS:HA	2.53	0.43
1:A:2034:ASP:OD2	1:A:2089:TYR:OH	2.33	0.43
1:E:822:LEU:HD22	1:E:1091:SER:HB2	2.00	0.43
1:E:1176:ARG:HH22	1:E:1281:ILE:HD12	1.82	0.43

Continued on next page...

CONFIDENTIAL VALIDATION REPORT

Continued from previous page...

Atom-1	Atom-2	Interatomic distance (Å)	Clash overlap (Å)
1:C:2471:VAL:HA	1:C:2474:VAL:HG12	2.00	0.43
1:A:2353:GLN:HB3	1:A:2366:VAL:HG22	2.01	0.43
1:A:2204:TRP:CH2	1:C:2021:LEU:HB3	2.53	0.43
1:C:2204:TRP:CH2	1:E:2021:LEU:HB3	2.53	0.43
1:E:2471:VAL:HA	1:E:2474:VAL:HG12	2.01	0.43
1:C:2064:ILE:HG22	1:C:2065:LEU:HD22	2.01	0.43
1:A:2188:LYS:NZ	1:C:2143:THR:O	2.48	0.43
1:C:2353:GLN:HB3	1:C:2366:VAL:HG22	2.00	0.43
1:E:996:LEU:HD11	1:E:1288:PHE:HD1	1.84	0.43
1:A:1521:ARG:HA	1:A:1528:ARG:HH22	1.84	0.43
1:A:996:LEU:HD11	1:A:1288:PHE:HD1	1.84	0.43
1:C:1079:TRP:HE3	1:C:1080:LEU:HD12	1.83	0.43
1:C:996:LEU:HD11	1:C:1288:PHE:HD1	1.84	0.43
1:E:1211:ASP:OD1	1:E:1294:ARG:NH1	2.52	0.43
1:C:942:ARG:O	1:C:946:HIS:ND1	2.45	0.43
1:E:2353:GLN:HB3	1:E:2366:VAL:HG22	2.00	0.43
1:E:1964:ASP:O	1:E:1968:THR:OG1	2.32	0.43
1:E:2312:THR:HA	1:E:2351:ARG:HH12	1.82	0.43
1:A:1211:ASP:OD1	1:A:1294:ARG:NH1	2.52	0.43
1:C:1211:ASP:OD1	1:C:1294:ARG:NH1	2.52	0.43
1:E:2286:ILE:O	1:E:2444:LEU:N	2.50	0.43
1:C:1521:ARG:HA	1:C:1528:ARG:HH22	1.84	0.42
1:C:822:LEU:HD22	1:C:1091:SER:HB2	2.00	0.42
1:C:2184:LYS:NZ	1:E:2146:THR:O	2.52	0.42
1:A:2064:ILE:HG22	1:A:2065:LEU:HD22	2.01	0.42
1:A:2471:VAL:HA	1:A:2474:VAL:HG12	2.00	0.42
1:E:2109:PHE:HA	1:E:2112:LYS:HE2	2.00	0.42
1:E:1521:ARG:HA	1:E:1528:ARG:HH22	1.84	0.42
1:C:2318:ARG:NH2	1:E:2245:GLN:O	2.52	0.42
1:C:2286:ILE:O	1:C:2444:LEU:N	2.50	0.42
1:A:2293:LEU:HD22	1:A:2429:TRP:HE1	1.85	0.42
1:E:2293:LEU:HD22	1:E:2429:TRP:HE1	1.85	0.42
1:E:2407:ARG:HG2	1:E:2428:GLU:HG2	2.02	0.42
1:E:2288:GLY:N	1:E:2442:ASN:O	2.51	0.42
1:E:2151:ASN:ND2	1:E:2526:TYR:OH	2.40	0.42
1:E:2535:SER:OG	1:E:2538:THR:OG1	2.25	0.42
1:A:1082:LEU:HD13	1:A:1082:LEU:HA	1.95	0.42
1:A:1963:HIS:HA	1:A:1966:LEU:HD12	2.01	0.42
1:C:2318:ARG:NH1	1:E:2244:GLN:OE1	2.53	0.42
1:C:802:ARG:HG2	1:C:944:GLN:HE22	1.85	0.41
1:E:1963:HIS:HA	1:E:1966:LEU:HD12	2.01	0.41

Continued on next page...

CONFIDENTIAL VALIDATION REPORT

Continued from previous page...

Atom-1	Atom-2	Interatomic distance (Å)	Clash overlap (Å)
1:A:2288:GLY:N	1:A:2442:ASN:O	2.51	0.41
1:C:2293:LEU:HD22	1:C:2429:TRP:HE1	1.85	0.41
1:E:1082:LEU:HA	1:E:1082:LEU:HD13	1.94	0.41
1:A:2286:ILE:O	1:A:2444:LEU:N	2.50	0.41
1:A:802:ARG:HG2	1:A:944:GLN:HE22	1.85	0.41
1:C:2535:SER:OG	1:C:2538:THR:OG1	2.25	0.41
1:E:2064:ILE:HG22	1:E:2065:LEU:HD22	2.01	0.41
1:E:2354:LEU:HD23	1:E:2357:LEU:HD12	2.03	0.41
1:A:1040:LEU:HD22	1:A:1101:LEU:HD23	2.03	0.41
1:E:1087:ARG:NH2	1:E:1257:GLY:O	2.54	0.41
1:E:802:ARG:HG2	1:E:944:GLN:HE22	1.85	0.41
1:A:2407:ARG:HG2	1:A:2428:GLU:HG2	2.02	0.41
1:C:1769:PHE:HA	1:C:1770:PRO:HD3	1.88	0.41
1:C:1963:HIS:HA	1:C:1966:LEU:HD12	2.01	0.41
1:C:2493:MET:HA	1:E:2534:ARG:HG2	2.03	0.41
1:E:2333:VAL:HG11	1:E:2382:PRO:HA	2.03	0.41
1:A:2479:LYS:HD3	1:C:2480:PHE:HE1	1.86	0.41
1:A:2151:ASN:ND2	1:A:2526:TYR:OH	2.40	0.41
1:C:851:CYS:O	1:C:854:THR:OG1	2.34	0.41
1:E:1253:CYS:SG	1:E:1254:THR:N	2.94	0.41
1:A:2497:LEU:HA	1:A:2498:PRO:HD3	1.92	0.41
1:C:2354:LEU:HD23	1:C:2357:LEU:HD12	2.03	0.41
1:C:1253:CYS:SG	1:C:1254:THR:N	2.94	0.41
1:E:2504:LEU:HA	1:E:2504:LEU:HD23	1.90	0.41
1:A:2378:ALA:HB3	1:A:2450:SER:HA	2.03	0.41
1:C:1087:ARG:NH2	1:C:1257:GLY:O	2.54	0.41
1:E:1040:LEU:HD22	1:E:1101:LEU:HD23	2.03	0.40
1:A:1716:LEU:HD22	1:A:2051:VAL:HG22	2.03	0.40
1:C:2238:LEU:HD22	1:C:2430:TRP:CE2	2.57	0.40
1:E:851:CYS:O	1:E:854:THR:OG1	2.34	0.40
1:A:1087:ARG:NH2	1:A:1257:GLY:O	2.54	0.40
1:A:2204:TRP:HH2	1:C:2021:LEU:HB3	1.86	0.40
1:C:2407:ARG:HG2	1:C:2428:GLU:HG2	2.02	0.40
1:E:822:LEU:HD13	1:E:1094:LEU:HB3	2.04	0.40
1:C:2226:VAL:O	1:C:2243:ALA:N	2.54	0.40
1:A:1253:CYS:SG	1:A:1254:THR:N	2.94	0.40
1:A:1214:ILE:HG12	1:A:1290:LEU:HB3	2.03	0.40
1:A:2226:VAL:O	1:A:2243:ALA:N	2.54	0.40
1:A:2360:GLY:HA2	1:A:2407:ARG:HH12	1.87	0.40
1:A:2333:VAL:HG11	1:A:2382:PRO:HA	2.03	0.40
1:A:2535:SER:OG	1:A:2538:THR:OG1	2.25	0.40

Continued on next page...

CONFIDENTIAL VALIDATION REPORT

Continued from previous page...

Atom-1	Atom-2	Interatomic distance (Å)	Clash overlap (Å)
1:A:823:LYS:HA	1:A:1092:THR:HG22	2.04	0.40
1:C:2308:LEU:HB3	1:C:2355:ALA:HB2	2.03	0.40
1:E:1214:ILE:HG12	1:E:1290:LEU:HB3	2.03	0.40
1:A:2129:PRO:HG3	1:E:2475:LEU:HD23	2.03	0.40

There are no symmetry-related clashes.

5.3 Torsion angles (i)

5.3.1 Protein backbone (i)

In the following table, the Percentiles column shows the percent Ramachandran outliers of the chain as a percentile score with respect to all PDB entries followed by that with respect to all EM entries.

The Analysed column shows the number of residues for which the backbone conformation was analysed, and the total number of residues.

Mol	Chain	Analysed	Favoured	Allowed	Outliers	Percentiles	
1	A	1478/2547 (58%)	1358 (92%)	120 (8%)	0	100	100
1	C	1478/2547 (58%)	1359 (92%)	119 (8%)	0	100	100
1	E	1478/2547 (58%)	1360 (92%)	118 (8%)	0	100	100
All	All	4434/7641 (58%)	4077 (92%)	357 (8%)	0	100	100

There are no Ramachandran outliers to report.

5.3.2 Protein sidechains (i)

In the following table, the Percentiles column shows the percent sidechain outliers of the chain as a percentile score with respect to all PDB entries followed by that with respect to all EM entries.

The Analysed column shows the number of residues for which the sidechain conformation was analysed, and the total number of residues.

Mol	Chain	Analysed	Rotameric	Outliers	Percentiles	
1	A	1198/2246 (53%)	1195 (100%)	3 (0%)	94	97
1	C	1198/2246 (53%)	1195 (100%)	3 (0%)	94	97
1	E	1198/2246 (53%)	1195 (100%)	3 (0%)	94	97

Continued on next page...

CONFIDENTIAL VALIDATION REPORT

Continued from previous page...

Mol	Chain	Analysed	Rotameric	Outliers	Percentiles	
All	All	3594/6738 (53%)	3585 (100%)	9 (0%)	94	97

All (9) residues with a non-rotameric sidechain are listed below:

Mol	Chain	Res	Type
1	A	903	ARG
1	A	1547	ARG
1	A	1971	ARG
1	C	903	ARG
1	C	1547	ARG
1	C	1971	ARG
1	E	903	ARG
1	E	1547	ARG
1	E	1971	ARG

Some sidechains can be flipped to improve hydrogen bonding and reduce clashes. All (12) such sidechains are listed below:

Mol	Chain	Res	Type
1	A	944	GLN
1	A	1013	HIS
1	A	1047	GLN
1	A	1669	GLN
1	A	2050	GLN
1	C	944	GLN
1	C	1013	HIS
1	C	1047	GLN
1	C	2050	GLN
1	E	944	GLN
1	E	1047	GLN
1	E	2050	GLN

5.3.3 RNA (i)

There are no RNA molecules in this entry.

5.4 Non-standard residues in protein, DNA, RNA chains (i)

There are no non-standard protein/DNA/RNA residues in this entry.

CONFIDENTIAL VALIDATION REPORT

CONFIDENTIAL VALIDATION REPORT

5.5 Carbohydrates (i)

There are no carbohydrates in this entry.

5.6 Ligand geometry (i)

There are no ligands in this entry.

5.7 Other polymers (i)

There are no such residues in this entry.

5.8 Polymer linkage issues (i)

There are no chain breaks in this entry.
